# Supplementary material for: The Increase of the Functional Entropy of the Human Brain with Age
Source: Sci Rep. 2013 Oct 9;3:2853. doi: 10.1038/srep02853 (PMC3793229; doi:10.1038/srep02853)
Supplement: Supplementary Information [file srep02853-s4.pdf]

The Increase of the Entropy of the Human Brain with Age.

Corresponding to
Jianfeng Feng

Department of Computer Science and Centre for Scientific Computing
Warwick University
Coventry CV4 7AL, UK
Email: jianfeng.feng@warwick.ac.uk

Information of other authors:

Y. Yao
Center for Computational Systems Biology, Fudan University
Shanghai 200433, PRC
Email: yyao@fudan.edu.cn

WL. Lu
Center for Computational Systems Biology, Fudan University
Shanghai 200433, PRC
Email: wenlian.lu@gmail.com

B. Xu
Center for Computational Systems Biology, Fudan University
Shanghai 200433, PRC
Email: bingxu1005@gmail.com

CB. Li
Department of Biological Psychiatry, Shanghai Mental Health Centre
Shanghai Jiao Tong University School of Medicine
Shanghai 200030, PRC
Email: chunbo_li@163.com

CP. Lin
Brain Connectivity Laboratory, Institute of Neuroscience
National Yang-Ming University
Taipei 11221, Taiwan
Email: cplin@ym.edu.tw

D. Waxman
Center for Computational Systems Biology, Fudan University
Shanghai 200433, PRC
Email: davidwaxman@fudan.edu.cn

Supplementary Information

Table of Contents

1 Detailed information of the samples from the FCON 1000 Project 2
2 Names and abbreviations of AAL brain regions 3
3 Entropy property proofs 4
3.1 Why the discrete entropy can take place of the relative entropy? 4
3.2 Entropy of the whole brain is not less than the average of every single brain region's entropy. 5
3.3 Entropy will grow higher with more intervals in [-1,1] 6
4 Entropy vs choice of atlas 7
4.1 Atlases with different numbers of brain regions 7
4.2 Atlases with a fixed number of brain regions 8
5 Functional entropy and the mean correlation coefficient 9
6 Functional entropy vs different running windows 11
7 Fucntional entropy in schizophrenia patients 12
8 Functional entropy in the left, right and inter hemispheres. 13
9 Increasing functional entropy in a single database 16
10 Age distribution of our dataset 18
11 Computational model of a brain network 19
11.1 Neuron dynamics 19
11.2 BOLD signal 20
11.3 Simulation of the functional entropy 21
12 Functional entropy before human maturity 27
13 Functional entropy with estrogen in females 28
14 Similar entropy gender pattern found in gray matter volume size 29
15 Results with flatting age distribution 31
16 Understanding the functional entropy: a mathematical description 32
17 References 34
18 Appendix: Matlab code of entropy 35

1 Detailed information of the samples from the FCON 1000 Project

Database	Quantity	Male/Female	Age (years)
Atlanta	27	12/15	29.9±8.6
Baltimore	23	8/15	29.3±5.5
Beijing_Zang	193	75/118	21.2±1.8
Berlin_Margulies	23	12/11	30.1±5.1
Cambridge_Buckner	192	71/121	21.1±2.3
Cleveland	31	11/20	43.5±11.1
Dallas	24	12/12	42.6±20.1
ICBM	36	15/21	38.2±17.6
Leiden_2180	10	10/0	23.4±2.5
Leiden_2200	19	11/8	21.7±2.6
Leipzig	37	16/21	26.2±5.0
Milwaukee_b	46	15/31	53.5±5.8
Munchen	11	6/5	66.6±3.4
NewHeaven_a	18	10/8	31.6±10.3
NewHeaven_b	16	8/8	26.9±6.3
Newark	18	9/9	24.3±4.0
Orangeburg	20	15/5	40.7±11.0
Oulu	22	7/15	21.3±0.6
PaloAlto	17	2/15	32.5±8.1
Pittsburgh	14	8/6	36.0±8.6
Queensland	18	11/7	26.3±3.7
Saintlouis	27	13/14	25.3±2.3
Whole Database	842	357/485	28.3±12.3

Table S1. Detailed information of 22 databases from the FCON 1000 Project

2 Names and abbreviations of AAL brain regions

Names and abbreviations of Regions Of Interest (ROIs).

**Region**	**Abbr.**	**Region**	**Abbr.**
Amygdala	AMYG	Orbitofrontal cortex (middle)	ORBmid
Angular gyrus	ANG	Orbitofrontal cortex (superior)	ORBsup
Anterior cingulate gyrus	ACG	Pallidum	PAL
Calcarine cortex	CAL	Paracentral lobule	PCL
Caudate	CAU	Parahippocampal gyrus	PHG
Cuneus	CUN	Postcentral gyrus	PoCG
Fusiform gyrus	FFG	Posterior cingulate gyrus	PCG
Heschl gyrus	HES	Precentral gyrus	PreCG
Hippocampus	HIP	Precuneus	PCUN
Inferior occipital gyrus	IOG	Putamen	PUT
Inferior frontal gyrus (opercula)	IFGoperc	Rectus gyrus	REC
Inferior frontal gyrus (triangular)	IFGtriang	Rolandic operculum	ROL
Inferior parietal lobule	IPL	Superior occipital gyrus	SOG
Inferior temporal gyrus	ITG	Superior frontal gyrus (dorsal)	SFGdor
Insula	INS	Superior frontal gyrus (medial)	SFGmed
Lingual gyrus	LING	Superior parietal gyrus	SPG
Middle cingulate gyrus	MCG	Superior temporal gyrus	STG
Middle occipital gyrus	MOG	Supplementary motor area	SMA
Middle frontal gyrus	MFG	Supramarginal gyrus	SMG
Middle temporal gyrus	MTG	Temporal pole (middle)	TPOmid
Olfactory	OLF	Temporal pole (superior)	TPOsup
Orbitofrontal cortex (inferior)	ORBinf	Thalamus	THA
Orbitofrontal cortex (medial)	ORBmed		

Table S2. Names and abbreviations of AAL brain regions

3 Entropy property proofs

3.1 Why the discrete entropy can take place of the relative entropy?

In the main text, the entropy is defined as the relative entropy[1]*, which is the KL (Kullback-Leibler) divergence from the correlation distribution to a reference measure (the Lebesgue measure)* (B). *In our work, this can also be considered as a differential entropy*[1]*. When calculating, however, we use an entropy appropriate to a discrete distribution (that results from binning the data). We shall refer to this as `the discrete entropy'. Here we give the reasons why we can use the discrete entropy to take the place of the relative entropy, the differential entropy, and does not lead to errors.*

Proof:

Let P and m denote the correlation distribution and the reference measure. We assume that $\mathrm{P(dx) = f(x)m(dx)}$. We then define B as the Lebesgue measure in $[-1,1]$ and μ_k as the counting measure that separate $[-1,1]$ evenly into k parts, with a sum of 2. Thus,

$$\mathrm{D}_{KL}(\mathrm{P}||\mathrm{B}) = -\int f_B(x)\log\big(f_B(x)\big)\,B(dx)$$

$$= -\int f_B(x)\log\big(f_B(x)\big)\,dx, \quad (1)$$

and

$$\mathrm{D}_{KL}(\mathrm{P}||\mu_k) = -\int f_B(x)\log\left(f_{\mu_k}(x)\right)\mu_k(dx)$$

$$= -\sum_{i=1}^{k}\frac{kP_i}{2}\log\left(\frac{kP_i}{2}\right)\times\frac{2}{k}, \quad (2)$$

where

$$\mathrm{P}_i = \int_{-1+(i-1)*2/k}^{-1+i*2/k} f_B(x)dx. \quad (3)$$

Since

$$-\sum_{i=1}^{k}\frac{kP_i}{2}\log\left(\frac{kP_i}{2}\right)\times\frac{2}{k} = -\sum_{i=1}^{k}P_i\log(P_i) - \log\left(\frac{k}{2}\right) \quad (4)$$

and $\log\left(\frac{k}{2}\right)$ is a constant, we need only calculate

$$-\sum_{i=1}^{k}P_i\log(P_i), \quad (5)$$

if we compare the difference of KL divergence $\mathrm{D}_{KL}(\mathrm{P}||\mu_k)$ with two different correlation distributions, P_1 and P_2. In addition, $-\sum_{i=1}^{k}P_i\log(P_i)$ is the discrete Shannon entropy[2]. As the μ_k will converge to B with k increasing, $\mathrm{D}_{KL}(\mathrm{P}||\mu_k)$ also converges to $\mathrm{D}_{KL}(\mathrm{P}||\mathrm{B})$ as k increases. Therefore, we can use the discrete entropy $-\sum_{i=1}^{k}P_i\log(P_i)$ to replace the relative entropy in our story without errors.

3.2 Entropy of the whole brain is not less than the average of every single brain region's entropy.

Method I: Calculate the entropy directly using all 4005 brain region pairs.
Method II: Calculate the entropy of every brain region first, then average them.
Method I calculates the entropy of the whole brain, while Method II calculates the average of every single brain's entropy.
The entropy obtained by Method I is not less than that of Method II.

Proof:

Let $[-1,1]$ be separated into n parts and let P_{ji} stand for the probability that the correlation of the pairs connecting the j'th brain region occurs in the i'th part of $[-1,1]$. Define $\mathrm{H(P)}$ as the entropy calculated by Method I. Thus,

$$\mathrm{H(P)} = \mathrm{H}\left(\frac{1}{90}\sum_{i=1}^{90} P_{1i}, \cdots, \frac{1}{90}\sum_{i=1}^{90} P_{ni}\right). \quad (6)$$

Since the entropy H is a concave function of its argument[3],

$$\mathrm{H}\left(\frac{1}{90}\sum_{i=1}^{90} P_{1i}, \cdots, \frac{1}{90}\sum_{i=1}^{90} P_{ni}\right) \geq \frac{1}{90}\sum_{i=1}^{90} H(P_{1i}, \cdots P_{ni}). \quad (7)$$

The right hand side of the last inequality is simply the entropy calculated by Method II, where every term in the sum is the entropy of a single brain region. Therefore, the entropy extracted by Method 1 cannot be not less than that of Method II. Moreover, equality only happens when all brain regions have the same entropy.

3.3 Entropy will grow higher with more intervals in [-1,1]

In the previous parts of this Supplementary Information, we separated $[-1,1]$ *into 20 intervals. If we separate it into more parts, the entropy will be larger.*

Proof:

According to the property of Shannon entropy,

$$\mathrm{H}(P_{11},\cdots,P_{m1},\cdots,P_{1n},\cdots,P_{mn}) = \mathrm{H}(P_{11}+\cdots+P_{m1},\cdots,P_{1n}+\cdots+P_{mn}) + \sum_{i=1}^{n}(P_{1i}+\cdots+P_{mi})H\left(\frac{P_{1i}}{\sum_{j=1}^{m}P_{ji}},\cdots,\frac{P_{mi}}{\sum_{j=1}^{m}P_{ji}}\right). \quad (8)$$

The left side of the last equality is the entropy with mn parts in $[-1,1]$, while the first term in the right side of the equality is the entropy with n parts. Moreover, the last term of the equality is not less than zero. Thus, the entropy with mn parts is larger than that with n parts. In other words, if there are more parts in $[-1,1]$, the entropy will be higher.

4 Entropy vs choice of atlas

4.1 Atlases with different numbers of brain regions

In the main text, the atlas we used was the AAL template, with 90 brain regions. We have investigated the relationship between atlas choice, the number of brain regions and the value of the functional entropy. We used different atlases with various numbers of brain regions on one sample, which was from a young healthy male, where the quality of the MRI was quite good. Figure S1 shows the entropy results from different atlases (red nodes) and a fit of the data by an exponential (blue line). The relationship between entropy and the number of brain regions, N, is $\mathrm{Entropy} = 1.64\mathrm{N}^{-0.337} + 3.305$. According to this equation, the entropy asymptotically approaches a constant as the number of brain regions tends to infinity. The limiting value of the functional entropy of this individual is 3.305.

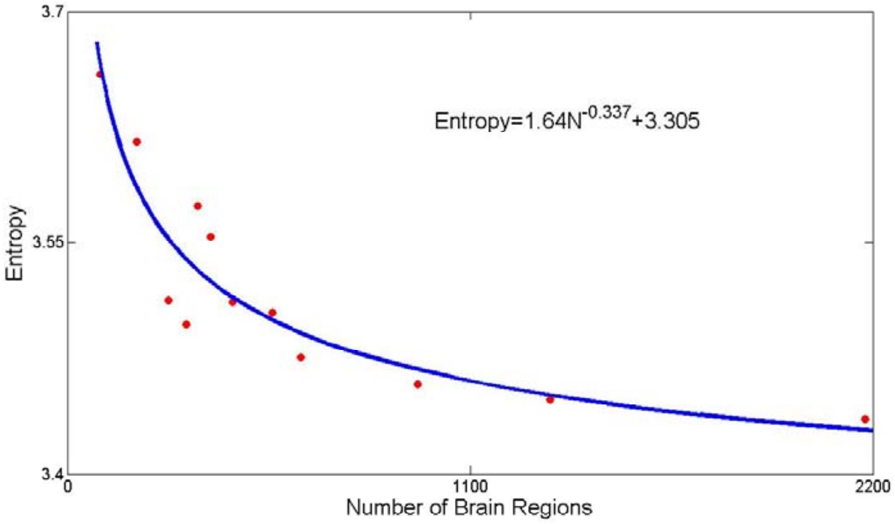

Figure S1
Entropy versus the number of brain regions. The red nodes are from an entropy calculation based on atlases with different numbers of brain regions. The blue line is a fit through the nodes from an exponential function.

Note that not all samples were suited to calculations of the entropy, using more than 2000 brain regions, because more brain regions lead to more noise. Thus, if we want to extract an accurate entropy with more brain regions, we need high quality MRI samples. Not all of the samples have sufficient quality, e.g., some samples are from 1.5T MRI or are affected by some head motions.

4.2 Atlases with a fixed number of brain regions

We further note that if we use different templates, but fix the number of brain regions, the results are quite stable. To see this consider Figure S2, where we have chosen the AAL atlas and separated it in different ways. We have considered 10 different atlases (all have 1024 brain regions) and extract the entropy of 20 normal young individuals, based on the various atlases. We have found no significant entropy differences for each sample, with various atlases. In Figure S2, different symbols label the entropy from different brain region atlases. The difference between the entropy from various atlases is sufficiently small that we need to amplify the scale to distinguish them. Thus, the entropy results are quite stable.

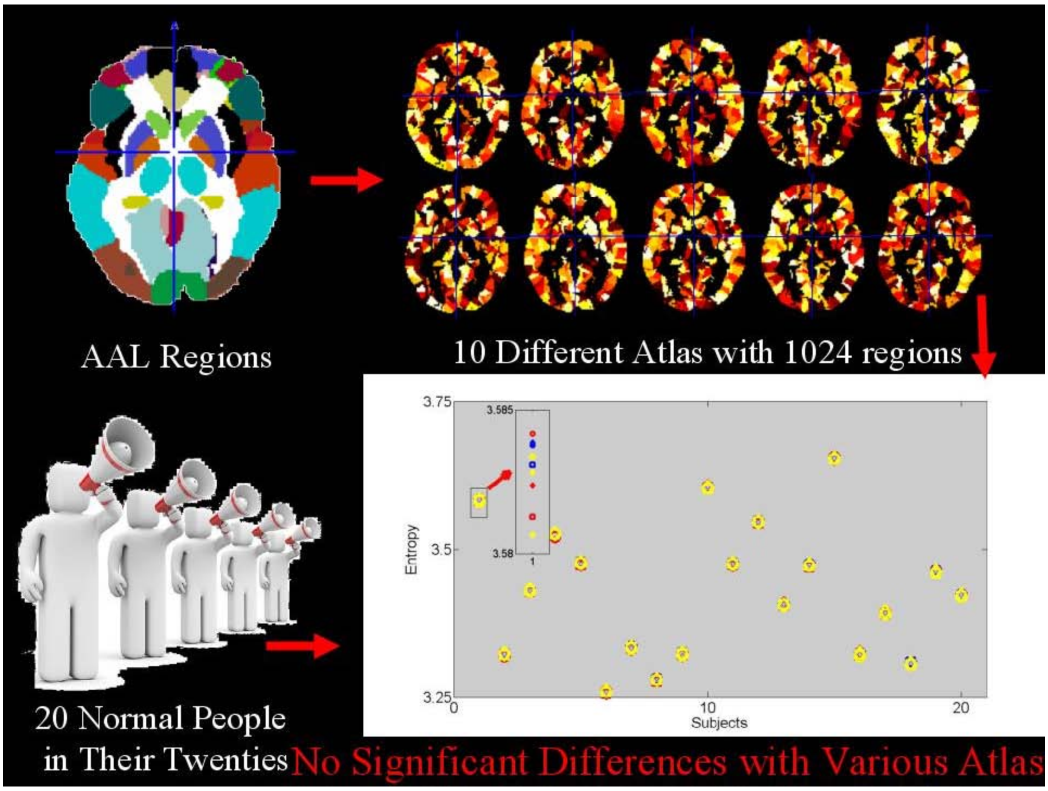

Figure S2
10 different atlases were chosen to separate the AAL atlas into more parts. We calculated the entropy in samples from 20 normal people with ages in the range 20 to 26. There are no significant differences with various atlases.

5 Functional entropy and the mean correlation coefficient

In the main text we introduced the idea of functional entropy. The functional entropy in our work can be considered as a kind of second order moment of the correlation coefficient distribution. In the main text, we have shown that the entropy is closely related to age. Originally, we used the mean correlation coefficient, as has been commonly used, and which can be considered as a first order moment of the correlation coefficient distribution. However, the mean correlation coefficient does not significantly change with age (results for this are shown in Figure S3). The data are similar to those we apply in the entropy study. The blue nodes are for males, the red ones for females. If we remove data of the youngest and oldest, say younger than 12 and older than 72, a plot of the mean correlation coefficient against age has a slope for both males and females, which is zero to five significant figures. The correlations between the mean correlation coefficient and age are -0.0537 for males and 0.0279 for females with p values of 0.2262 and 0.5118 respectively (Student's t-distribution for the Pearson correlation coefficient, with 610 and 634 degrees of freedom, respectively). Thus the first order moment appears to play no role in a study of ageing. We thus considered a second order moment, namely the functional entropy in our investigations.

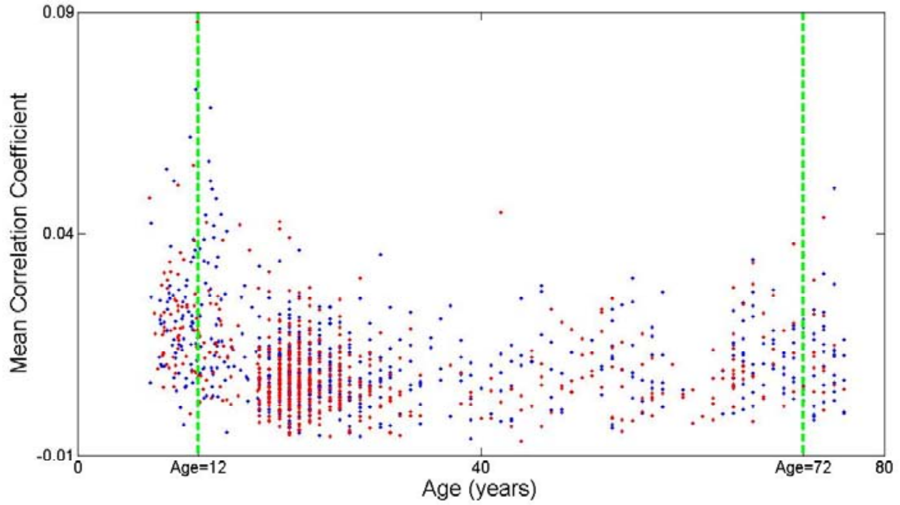

Figure S3

Mean correlation coefficient versus age. The red nodes are from females while blue nodes come from males.

Moreover, brain signals are found to demonstrate increased variability with age[4, 5]. We note that information entropy has a large value when there is a high level of randomness[6] and higher randomness is equivalent higher variability[7]. The above suggests that the notion of entropy will be useful in investigating the

neurobiology of ageing. Thus, we have focused on the functional entropy of the brain in the present study.

6 Functional entropy vs different running windows

In the main text, we used an age window of 25 years to determine Fig. 2b. Here, we demonstrate that choosing windows from 19 to 30 years do not significantly affect any conclusions we draw. In Figure S4, we show the results with an age window ranging from 19 to 30 years. We conclude that the results are stable and not greatly affected by the age window size. The crossover age is 50 years in all panels.

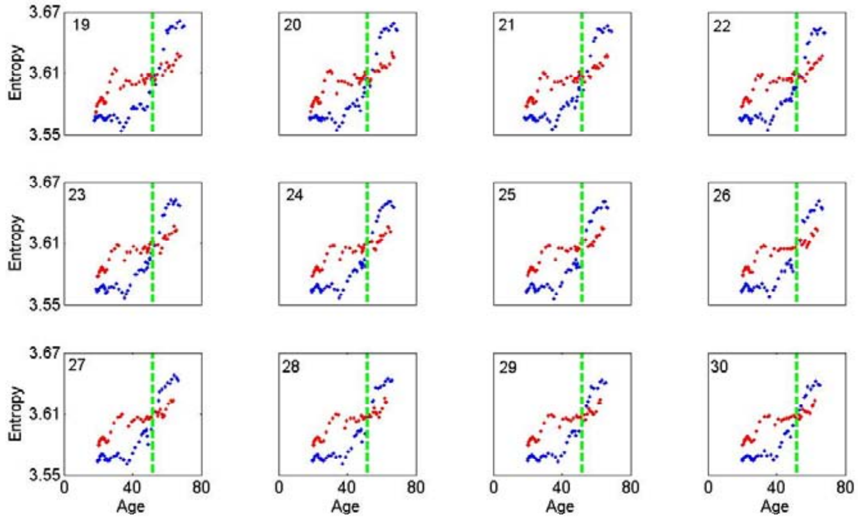

Figure S4

The running average of the functional entropy, with averaging performed over differently sized age windows. The numbers in the upper left corners of each figure are the size of the window, in years.

7 Fucntional entropy in schizophrenia patients

In the main text, we focused on normal individuals. Additionally, we can determine the functional entropy of individuals with mental disorders, such as schizophrenia. To proceed, we selected a sample from schizophrenia patients, with a mean age of 24 years. To compare the differences between normal people and schizophrenia patients, we selected another two groups from our original dataset. One is from normal people with a mean age of 24 years. The other is from elderly people with a mean age of 69 years. The distribution of all the three groups, by gender, is approximately half males and half females. We list the correlation coefficient distributions in Figure S5, where blue represents normal young individuals, red represents schizophrenia patients and white shows elderly people. It can be seen that the distribution of correlation coefficients for schizophrenia patients is narrower than that of normal people, while the distribution of correlation coefficients of the elderly people is broader, as claimed in the main text. In the language of functional entropy, the schizophrenia patients have lower entropy while the elderly people have higher entropy. As shown in some surveys[8], older people have a low chance of getting schizophrenia. The entropy difference between elderly people and schizophrenia patients may be the origin of this phenomenon, if the entropy difference between different classes of individuals plays the role of a risk difference. This point will be discussed in detail elsewhere.

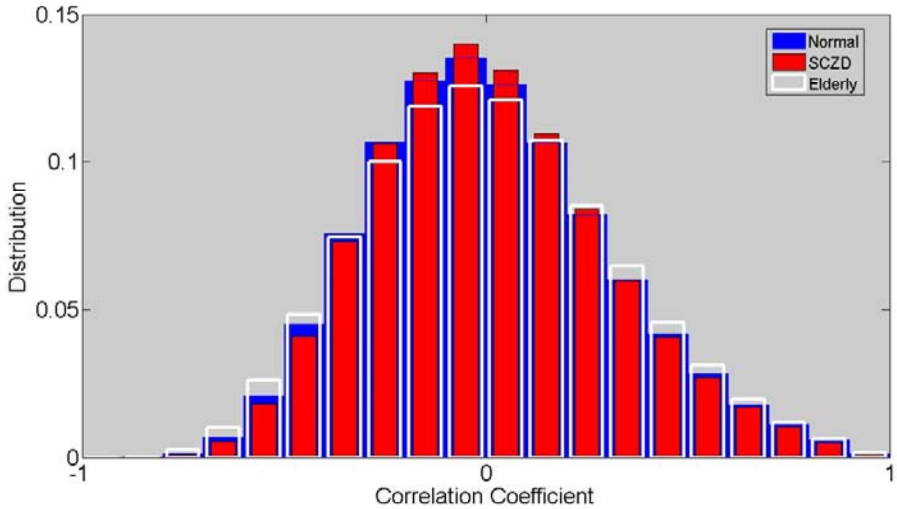

Figure S5

A histogram of the correlation coefficients of three groups of individuals: normal young (blue), normal elderly (white) and schizophrenia patients, denoted SCZD (red). The groups of normal young individuals and schizophrenia patients both have a mean age of 24 years, while the mean of the elderly group is 69 years. The gender distribution of the three groups is approximately half males and half females.

8 Functional entropy in the left, right and inter hemispheres.

In the main text, we focused on the functional entropy, based on the whole brain (from all 4005 pairs of different brain regions). Moreover, we can consider the functional entropy in the inter-hemisphere and intra-hemisphere. Similarly, we also apply the AAL atlas with 90 brain regions. Thus, in the left and right hemisphere, we have 990 (C_{45}^{2}) pairs, while there exist 2025 (45x45) pairs in the inter-hemisphere connections. Using the same calculational method for the functional entropy, we have also determined the functional entropy in the left, right and inter hemispheres. As shown in Figure S6 (left-hemisphere), Figure S7 (right-hemisphere) and Figure S8 (inter hemisphere), we can extract similar results to the ones based on whole brain entropy. The functional entropy in the left, right and inter hemisphere will also increase with ageing with a crossover at age 50. In addition, the functional entropy based on the inter hemisphere is lower than that based on the intra hemisphere, since the inter hemispheric functional and physical connections are lower than those of the intra hemisphere, this leads to lower correlation coefficients in the inter hemisphere. Moreover, the correlation between the functional entropy based on the inter hemisphere and age are lower than ones based on the intra hemisphere.

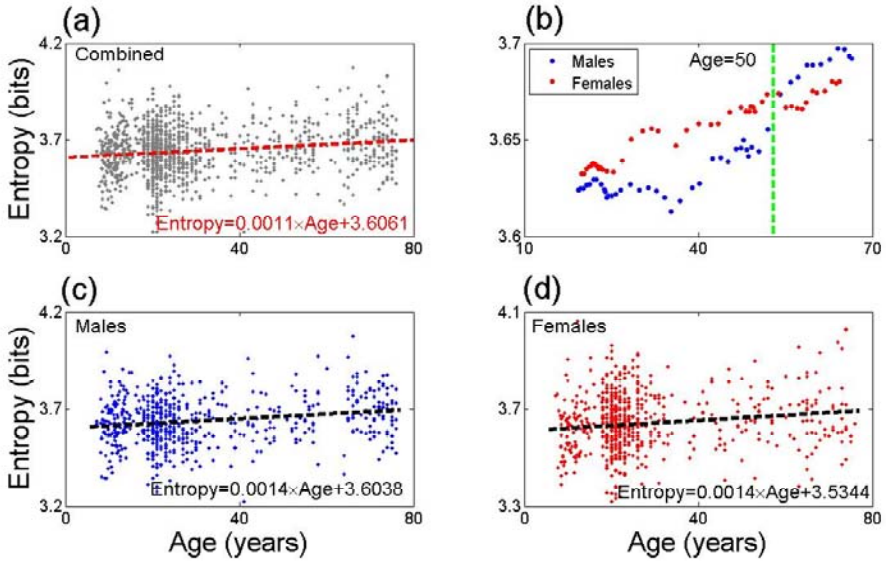

Figure S6 Functional entropy (of left hemisphere) vs age.

Panel (a): Functional entropy (of left hemisphere) of individuals versus their age (averaged over males and females). A mean increase of the entropy of 0.0011 bits per year was found from the data. Panel (b): A plot of the running average of the entropy, with a window of width 25 years, vs age. Note that there is a crossover in the male/female

entropies in the vicinity of 50 years. Panels (c) and (d): Entropy vs age of males and females with rates of increase of 0.0014 and 0.0014 bits per year, respectively. For the combined male and female groups, the linear correlation between entropy and age is statistically significant as indicated by their p values (1.29×10^{-9}, 4.02×10^{-12} and 5.41×10^{-11}, using Student's t-distribution for the Pearson correlation coefficient, degrees of freedom: 1246, 610 and 634, respectively).

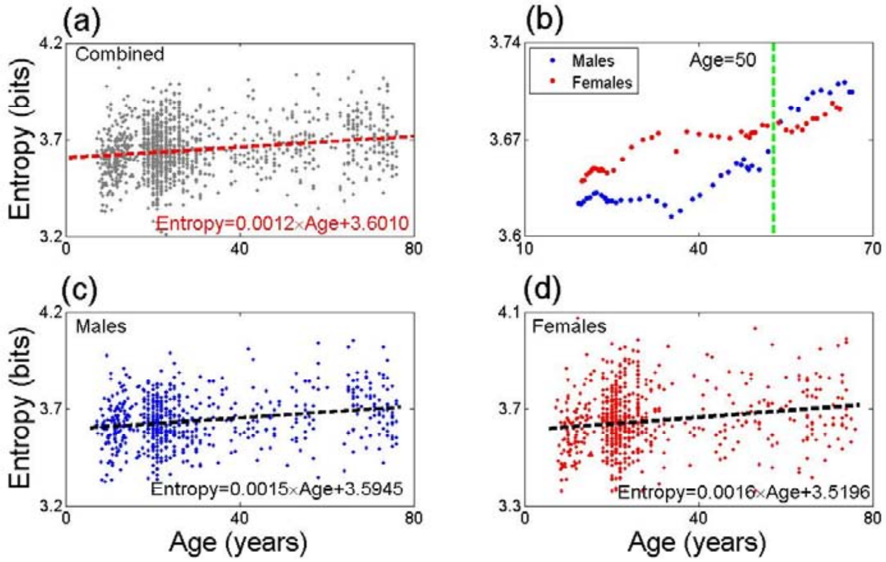

Figure S7 Functional entropy (of right hemisphere) vs. age.

Panel (a): Functional entropy (of right hemisphere) of individuals vs their age (averaged over males and females). An average increase of the entropy of 0.0012 bits per year was found from the data. Panel (b): A plot of the running average of the entropy, with a window of width 25 years vs age. Note that there is a crossover in the male/female entropies in the vicinity of 50 years. Panels (c) and (d): Entropy vs age of males and female with rates of increase of 0.0015 and 0.0016 bits per year, respectively. For the combined male and female group, the linear correlation between entropy and age is statistically significant as indicated by their p values (1.20×10^{-6}, 2.27×10^{-8} and 2.57×10^{-9}, using Student's t-distribution for the Pearson correlation coefficient, degrees of freedom, 1246, 610 and 634, respectively).

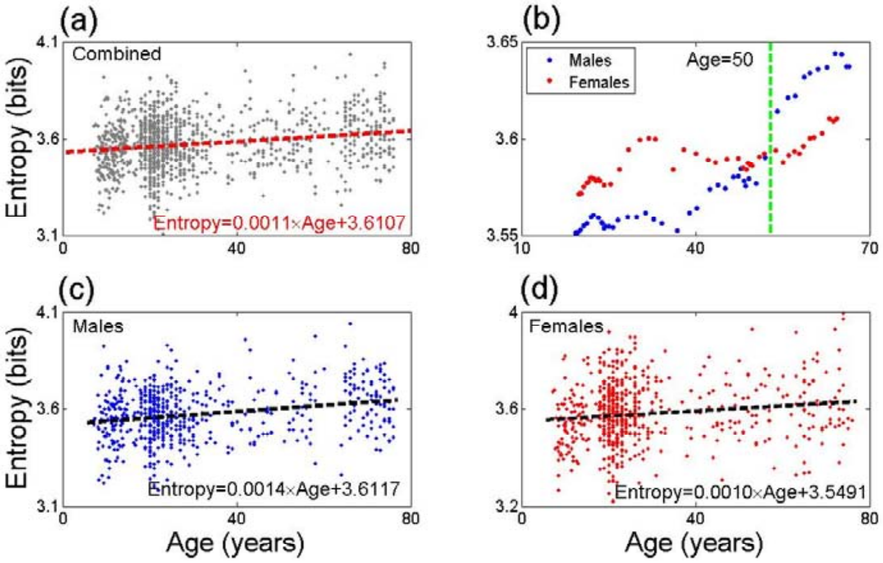

Figure S8 Functional entropy (of inter hemisphere) vs age.

Panel (a): Functional entropy (of inter hemisphere) of individuals vs their age (averaged over males and females). An average increase of the entropy of 0.0011 bits per year was found from the data. Panel (b): A plot of the running average of the entropy, with a window of width 25 years vs age. Note that there is a crossover in the male/female entropies in the vicinity of 50 years. Panels (c) and (d): Entropy vs age of males and female with an rate of increase of 0.0014 and 0.0010 bits per year, respectively. For the combined male and female group, the linear correlation between entropy and age is statistically significant as indicated by their p values (1.93×10^{-4}, 1.45×10^{-5e} and 9.06×10^{-4}, using Student's t-distribution for the Pearson correlation coefficient, degrees of freedom: 1246, 610 and 634, respectively).

9 Increasing functional entropy in a single database

In the main text, we combined 26 databases together to obtain an increase of functional entropy with age. To remove effects of any differences between different databases, we have carried out tests, such as removing some databases, to check the robustness of our results. We obtained very similar results, including an increase of functional entropy with age. Additionally, the crossover at age 50 persists. Additionally, we can find the increase functional entropy in the data from a *single* database. In our dataset, there exist two databases covering an age range of more than 50 years. They are the ICBM and Taiwan databases. As shown in Figure S9 and Figure S10, the data in the two databases also show an increasing functional entropy with age. Since the sample numbers of the two databases are 36 and 48, the correlations between the functional entropy and age are not statistically significant. The results for these databases partially certify the notion of increasing functional entropy with age.

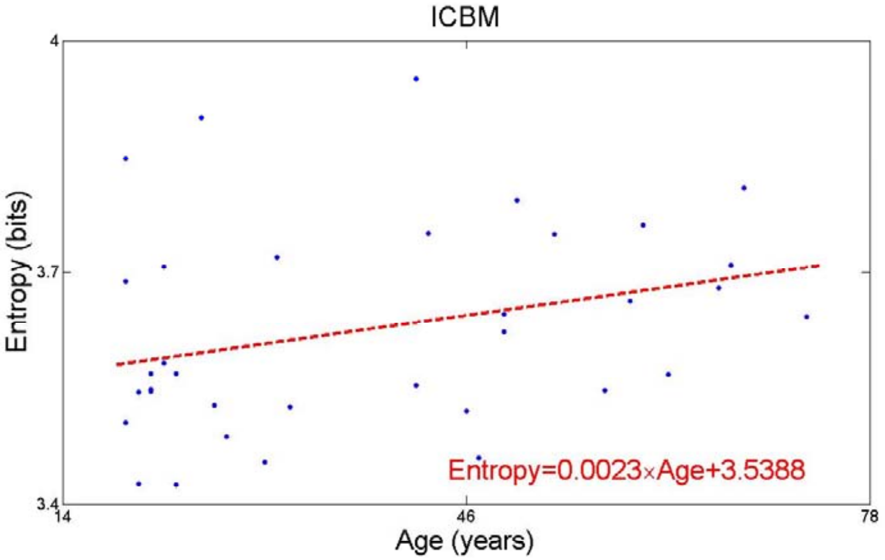

Figure S9 Functional entropy of individuals versus their age from ICBM database

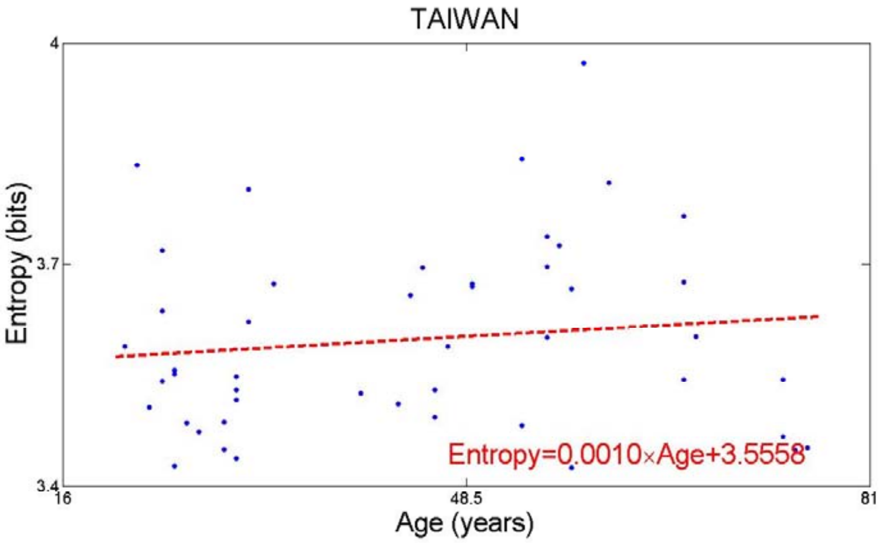

Figure S10 Functional entropy of individuals vs their age from the Taiwan database

10 Age distribution of our dataset

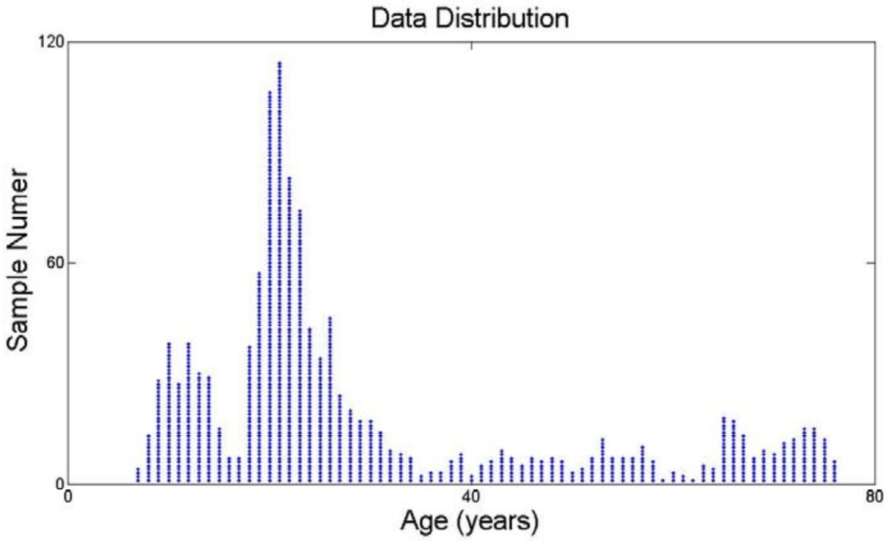

Figure S11 Age distribution of our datasets.

The sample number shows the number of samples with the same age. Most samples are from people in their twenties. Moreover, we have at least one sample in every year from 6 to 76 years old.

11 Computational model of a brain network

In the main text, we used a computational model from Deco et al's work[9] to verify our result about functional entropy.

11.1 Neuron dynamics

The global structure of the model is illustrated in Figure S12. Every brain region served as a node in a large scale network, which consists of a population of excitatory pyramidal neurons and a population of GABAergic inhibitory neurons, which are all-to-all connected. The communication between every two nodes is through synaptic connections between excitatory neurons in those areas.

For each brain area, an integrate-and-fire neuron model with excitatory (AMPA and NMDA) and inhibitory (GABA) synaptic receptors was applied. The dynamics of the membrane potential $\mathrm{V(t)}$ are described by:

$$C_m \frac{dV(t)}{dt} = -g_m(V(t) - V_L) - I_{syn}(t), \quad \text{if } V(t) < V_{thr}$$

$$V(t) = V_{reset}, \quad \text{if } V(t) \geq V_{thr} \qquad (9)$$

The total synaptic current I_{syn} is given by the sum of glutamatergic AMPA external background excitatory currents ($I_{AMPA,ext}$), AMPA ($I_{AMPA,rec}$) and NMDA ($I_{NMDA,rec}$) recurrent excitatory currents, and GABAergic recurrent inhibitory currents (I_{GABA}):

$$I_{syn} = I_{AMPA,ext} + I_{AMPA,rec} + I_{NMDA,rec} + I_{GABA}, \qquad (10)$$

where

$$I_{AMPA,ext}(\mathrm{t}) = g_{AMPA,ext}(\mathrm{V(t)} - V_E) \sum_j s_j^{AMPA,ext}(t),$$

$$I_{AMPA,rec}(\mathrm{t}) = g_{AMPA,rec}(\mathrm{V(t)} - V_E) \sum_j \omega_j^{AMPA} s_j^{AMPA,rec}(t),$$

$$I_{NMDA,rec}(\mathrm{t}) = \frac{g_{NMDA,rec}(\mathrm{V(t)} - V_E)}{1 + \gamma e^{-\beta V(t)}} \sum_j \omega_j^{NMDA} s_j^{NMDA,rec}(t),$$

$$I_{GABA}(\mathrm{t}) = g_{GABA}(\mathrm{V(t)} - V_I) \sum_j \omega_j^{GABA} s_j^{GABA}(t). \qquad (11)$$

The gating variables $s_j\ (t)$ are the fractions of open channels of neurons and are given by:

$$\frac{ds_j^{AMPA,ext}(t)}{dt} = -\frac{s_j^{AMPA,ext}(t)}{\tau_{AMPA}} + \sum_k \delta(t - t_j^k),$$

$$\frac{ds_j^{AMPA,rec}(t)}{dt} = -\frac{s_j^{AMPA,rec}(t)}{\tau_{AMPA}} + \sum_k \delta(t - t_j^k),$$

$$\frac{ds_j^{NMDA,rec}(t)}{dt} = -\frac{s_j^{NMDA,rec}(t)}{\tau_{NMDA,decay}} + \alpha x_j^{NMDA,rec}(t)\left(1 - s_j^{NMDA,rec}(t)\right),$$

$$\frac{dx_j^{NMDA,rec}(t)}{dt} = -\frac{x_j^{NMDA,rec}(t)}{\tau_{NMDA,rise}} + \sum_k \delta(t - t_j^k),$$

$$\frac{ds_j^{GABA}(t)}{dt} = -\frac{s_j^{GABA}(t)}{\tau_{GABA}} + \sum_k \delta(t - t_j^k), \tag{12}$$

The sums over the *k* index represent all of the spikes emitted by presynaptic neuron j (at times t_j^k). The description and value of most parameters are shown in Table S3.

Each local area contains 100 excitatory neurons and 100 inhibitory neurons. The connection strengths between and within the populations are determined by dimensionless strength ω. Illustrated in Figure S12, there are 4 different intra-connection strength: ①excitation (AMPA and NMDA) within excitatory neurons ω^1;② excitation (AMPA and NMDA) from excitatory neuron to inhibitory neuron $\omega^2 = 1$; ③ inhibition (GABA) from inhibitory neuron to excitatory neuron $\omega^3 = 1$; ④ inhibition (GABA) within inhibitory neurons $\omega^4 = 1$. We vary ω^1 systematically to see the implications for the global functional entropy. The inter-regional connection strength ω^{inter} is proportional to number of fibers linking every two regions. The neuroanatomical matrix whose element is fiber number, is obtained by Diffusion Tensor Imaging. Here, we used averaged structural matrix from 46 healthy people, which is showed in Figure S13.

All neurons always received an external background input from $N_{ext} = 800$ external neurons emitting independent Poisson spike trains at a rate of 3 Hz. More specifically, for all neurons inside a given population p, the resulting global spike train, which is still Poissonian, had a time-varying rate $v_{ext}^p(t)$, governed by

$$\tau_n \frac{dv_{ext}^p(t)}{dt} = -(v_{ext}^p(t) - v_0) + \sigma_v\sqrt{2\tau_n}\xi^p(t), \tag{13}$$

where $\tau_n = 300ms, v_0 = 2.4\ kHz, \sigma_v = 0.2$ is the standard deviation of $v_{ext}^p(t)$, and $\xi^p(t)$ is normalized Gaussian white noise

.

11.2 BOLD signal

The simulation of the fMRI BOLD signal is computed by means of the Balloon-Windkessel hemodynamic model[10]. The BOLD-signal of each region is driven by the level of neuronal activity summed over all neurons in both populations (excitatory and inhibitory populations) in that particular region. In all our simulations, neuronal activity is given by the rate of spiking activity in a time window of 1 ms. In brief, for the i'th region, neuronal activity z_i causes

an increase in a vasodilator signal s_i that is subject to autoregulatory feedback. Inflow f_i responds in proportion to this signal with concomitant changes in blood volume v_i and deoxyhemoglobincontent q_i. The equations relating these biophysical variables are:

$$\frac{ds_i(t)}{dt} = \varepsilon_i z_i - k_i s_i - \gamma_i(f_i - 1),$$

$$\frac{df_i(t)}{dt} = s_i,$$

$$\tau_i \frac{dv_i(t)}{dt} = f_i - v_i^{1/\alpha},$$

$$\tau_i \frac{dq_i(t)}{dt} = \frac{f_i(1-(1-\rho_i)^{1/f_i})}{\rho_i} - \frac{q_i v_i^{1/\alpha}}{v_i}, \quad (14)$$

where ρ is the resting oxygen extraction fraction. The BOLD signal is taken to be a static nonlinear function of volume and deoxyhemoglobin that comprises a volume-weighted sum of extra- and intravascular signals:

$$y_i = V_0\left(7\rho_i(1 - q_i) + 2\left(1 - \frac{q_i}{v_i}\right) + (2\rho_i - 0.2)(1 - v_i)\right), \quad (15)$$

where $V_0 = 0.02$ is the resting blood volume fraction. The biophysical parameters were taken as $\varepsilon_i = 0.2, k_i = 0.65, \gamma_i = 0.41, \tau_i = 0.98, \alpha = 0.32, \rho_i = 0.34$.

11.3 Simulation of the functional entropy

After we obtained the simulated BOLD time series, the global signal (average over all regions) was regressed out. Figure S14 shows typical temporal evolution of the simulated BOLD signal (after regression) for several brain regions. We then calculated the simulated functional connectivity by calculating the correlation matrix of the BOLD time series. Figure S15 and S16 plot an example of stimulated functional connectivity matrix and corresponding distribution of correlation, respectively. Using the calculation method of functional entropy, we could compare the simulated functional entropy with that from fMRI data.

When we increase intra-excitatory connection strength ω^1 with other parameters fixed, the firing rate of excitatory neurons in the whole brain increase. (Firing-rate amplification of inhibitory neurons can be ignored compared to that of excitatory neurons.) Based on the fact that the firing rate of one excitatory neuron, in resting state, is about $3\ \mathrm{Hz}$ and that the model of one neuron here could also described the dynamics of several neurons or a neuron mass, we can calculate the actual excitatory neuron number in each brain region by the 100 times mean firing rate divided by $3\mathrm{Hz}$. (Here we just use the averaged firing rate of all excitatory neurons in the whole brain, not in each brain region.) Figure S17 illustrates the positive correlation between actual excitatory neuron number and intra-excitatory connection strength. The

two red dashed lines show the range of connection strength [1.78,1.81], which makes the corresponding entropy match the human data. Based on the least squares line (black dashed line), the neuron number range is limited to [888,1130] (indicated by green text arrows).

		Excitatory neuron	Inhibitory neuron	Synapse	
Membrane capacitance C_m		0.5 nF	0.2 nF	Excitatory reversal potential V_E	0 mV
Leak conductance g_m		25 nS	20 nS	Inhibitory reversal potential V_I	−70 mV
Resting potential V_L		−70 mV	−70 mV	Decay time τ_{AMPA}	2 ms
Threshold potential V_{thr}		−50 mV	−50 mV	Rise time $\tau_{NMDA,rise}$	2 ms
Reset potential V_{reset}		−55 mV	−55 mV	Decay time $\tau_{NMDA,decay}$	100 ms
Refractory time τ_{ref}		2 ms	1 ms	Decay time τ_{GABA}	10 ms
Synaptic conductance	$g_{AMPA,ext}$	2.496 nS	1.944 nS	α	0.5 kHz
	$g_{AMPA,rec}$	0.104 nS	0.081 nS	β	0.062
	$g_{NMDA,rec}$	0.327 nS	0.258 nS	γ	0.28
	g_{GABA}	2.45 nS	1.2 nS		

Table S3.Neural and synaptic parameters

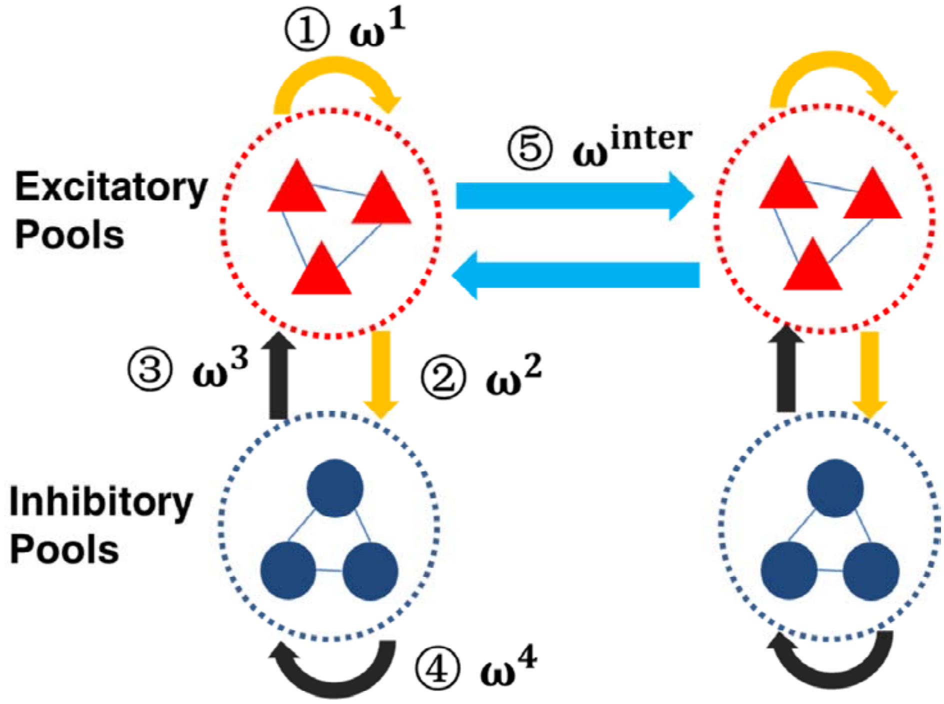

Figure S12
Schematic representation of the brain network. Each brain area is comprised of excitatory neurons (red triangles) and inhibitory interneurons (blue circles). ①②③④ represent the four different intra-connection during each brain area, and ⑤ describes the inter-connection between different brain area, which depends on DTI.

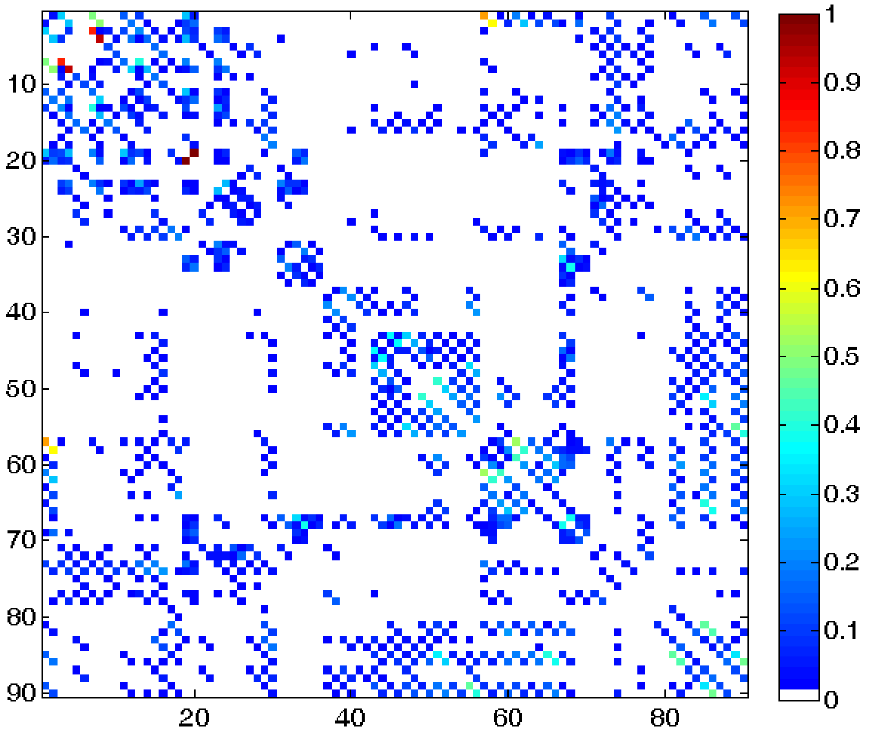

Figure S13

Neuroanatomical connectivity matrix, obtained by DTI after averaging across 46 human subjects.

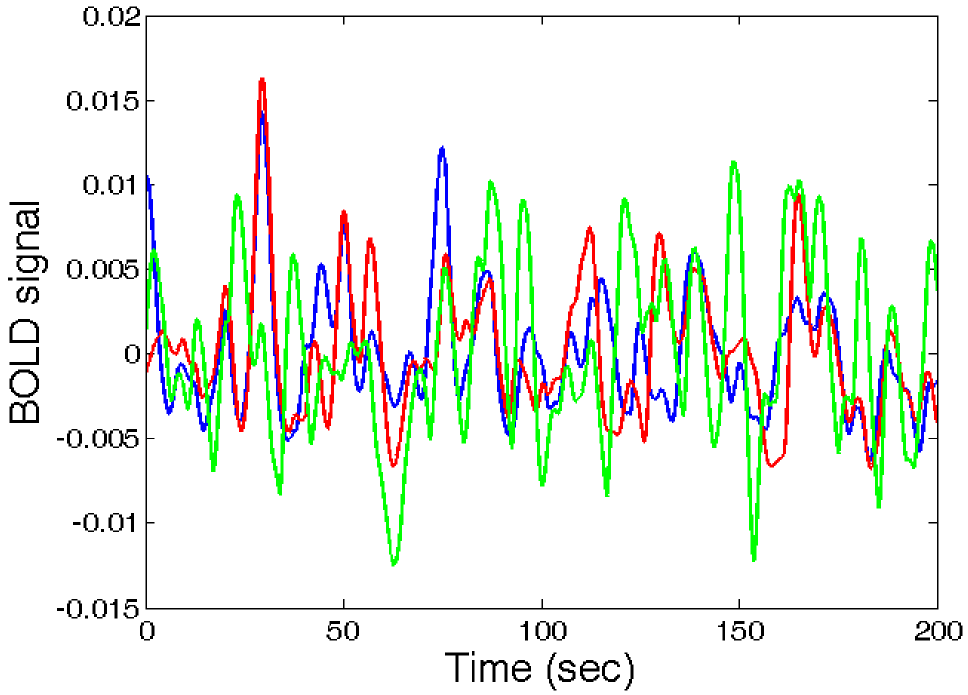

Figure S14

Simulated BOLD signal for thalamus (blue), Inferior temporal gyrus (red) and Insula(green) when intra-excitatory connection strength $\omega^1 = 1.81$.

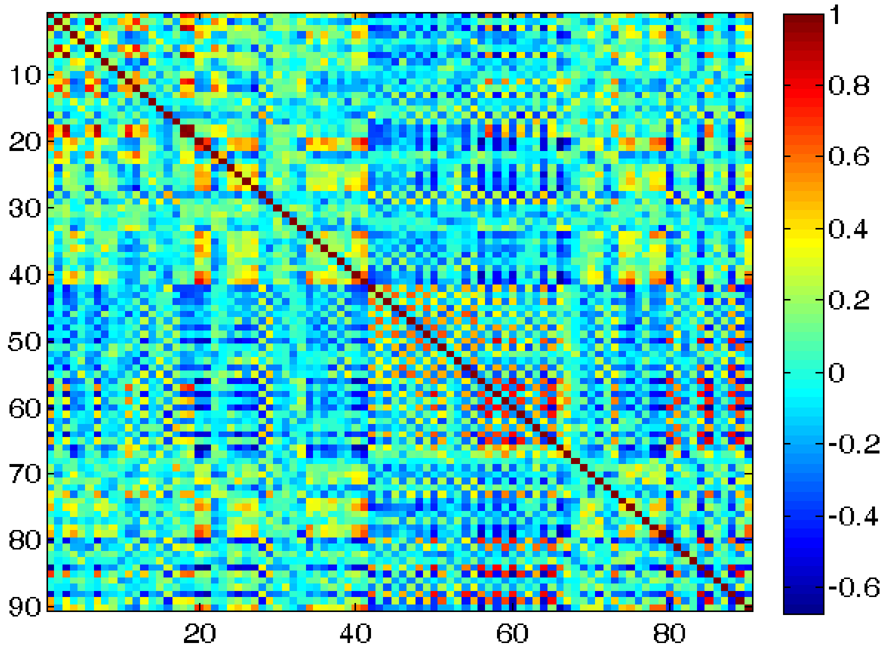

Figure S15

Simulated functional connectivity matrix when intra-excitatory connection strength $\omega^1 = 1.81$.

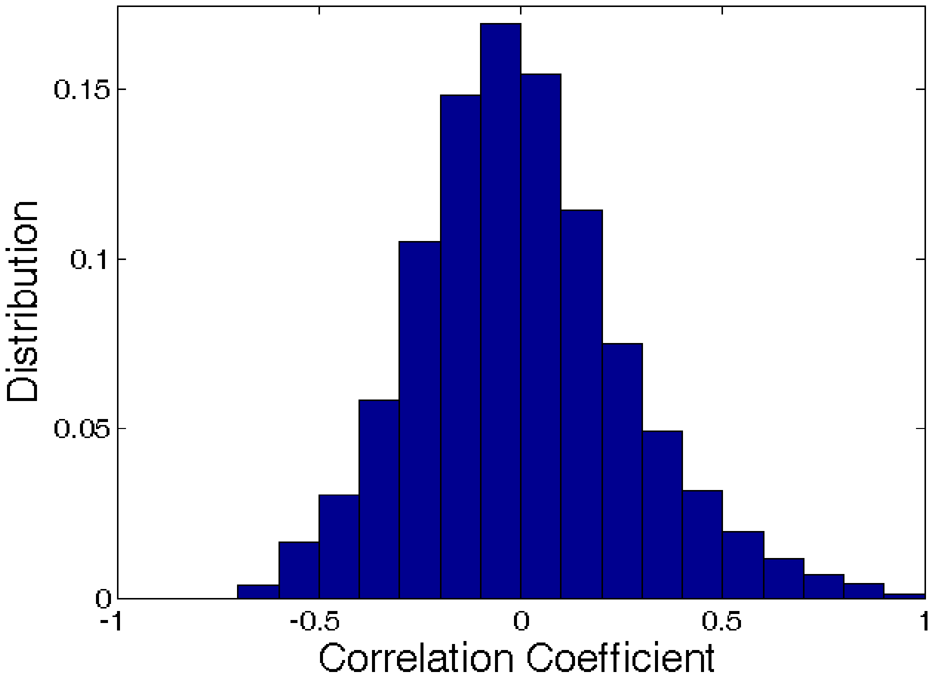

Figure S16

The distribution of correlation coefficients when the intra-excitatory connection strength $\omega^1 = 1.81$.

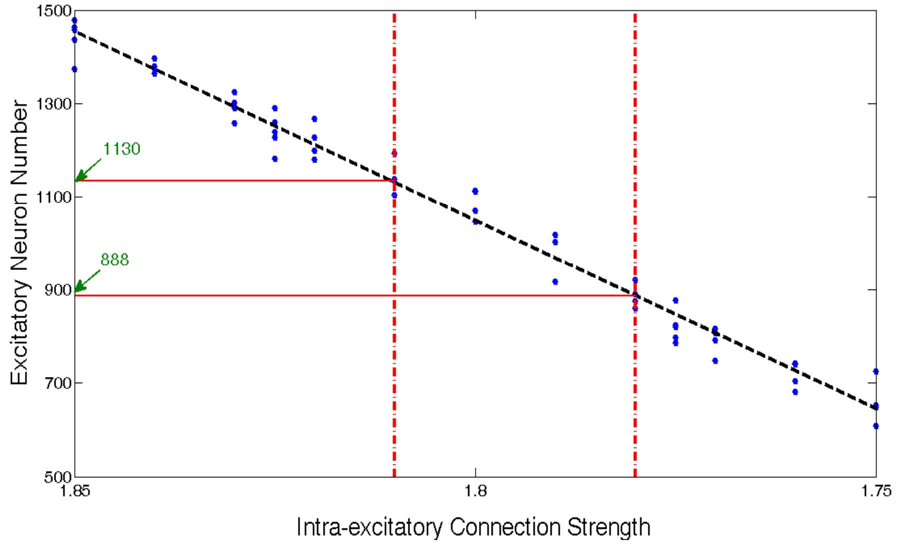

Figure S17

Excitatory neuron number versus intra-excitatory connection strength ω^1.

12 Functional entropy before human maturity

In this part, we focus on the increase functional entropy bit per year before human maturity. As shown in Figure S18, the increase functional entropy bit before 25 years old are higher than that during 25 and 50 years old in both males and females. For males, the increase functional entropy bit per year is 0.0014 bit/year before 25 years old and 0.0004 bit/year during 25 and 50 years old, while the females hold 0.0023 bit/year and 0.0000 bit/year. In conclusion, functional entropy grows higher during childhood and adolescence, and the increasing functional entropy rate will become lower after maturity.

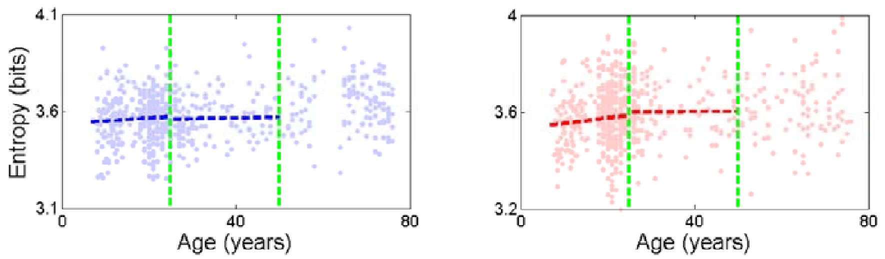

Figure S18

The figure represents the functional entropy trend with ageing. The left panel is from males, while the right one comes from females. The two green dashed lines in each panel stand for age 25 and 50.

13 Functional entropy with estrogen in females

In this part, we consider the relationship with the functional entropy and the estrogen. Other studies[11, 12] have reported that estrogen protects brain, so we compare the functional entropy trend before and after menarche. Since the average of menarche is 13 years old[13], we separate the samples before menopause into two groups, before and after 13 years old. As shown in Figure S19, females before 13 years old hold a higher increasing functional entropy. In particular, functional entropy increases 0.0118 bits per year before 13, but 0.0007 bit/year after that. This may be related to the estrogen level in females, which implies that estrogen protects brain.

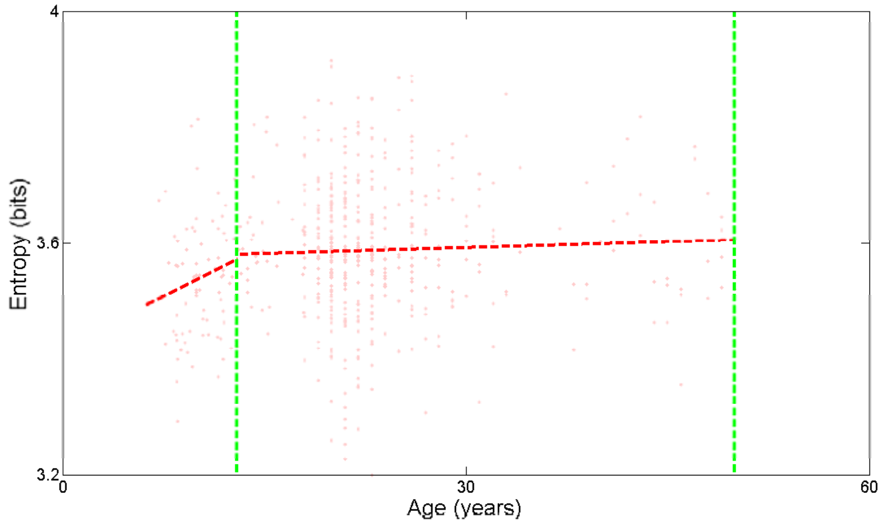

Figure S19

The figure represents the functional entropy trend with ageing in females. The two green dashed lines stand for age 13 and 50.

14 Similar entropy gender pattern found in gray matter volume size

We have 496 samples (252 males/ 244 females) with T1-image data. We applied the Voxel-based morphometry[14] (VBM8) to extract the gray matter size of all the AAL brain regions. As shown in Figure S20, the gray matter size will decrease with ageing in both males and females. Now we calculate the decrease cubic millimeter per year by linear regression analysis. Since 50 years old is a quite important threshold in the main text, we separate the samples into two groups, lower and older than 50 years old. For males, the gray matter size decreases 1620 mm^3 per year before 50 and 3090 mm^3 per year after 50, while that of females decreases 1930 mm^3 per year before 50 and 2960 mm^3 per year after 50. The decrease size will be larger in the group after 50 than that before 50 no matter in males or females. It should be emphasized that the decrease size per year in females is larger than that in males before 50, while this will go to the opposite direction after 50 years old. This is quite similar with the pattern of the entropy in males and females.

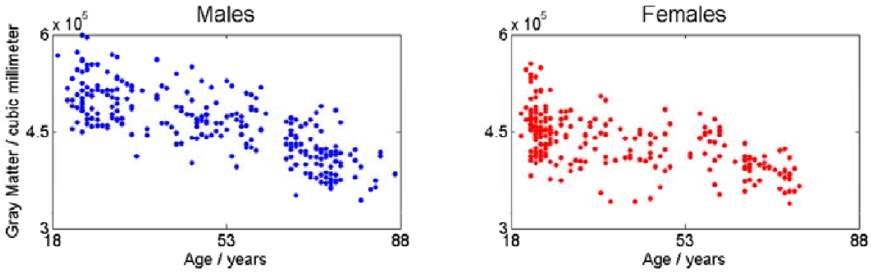

Figure S20

The figure represents the gray matter size trend with ageing. The left panel is from males, while the right one comes from females.

15 Results with flatting age distribution

As shown in Section 10, the age distribution of the dataset in this paper is not flat, which may leads to some errors. Thus, we randomly selected some subjects with flatting age distribution (six individuals per age), and found that the significant results were still present, functional entropy increased with age, as shown in Figure S21.

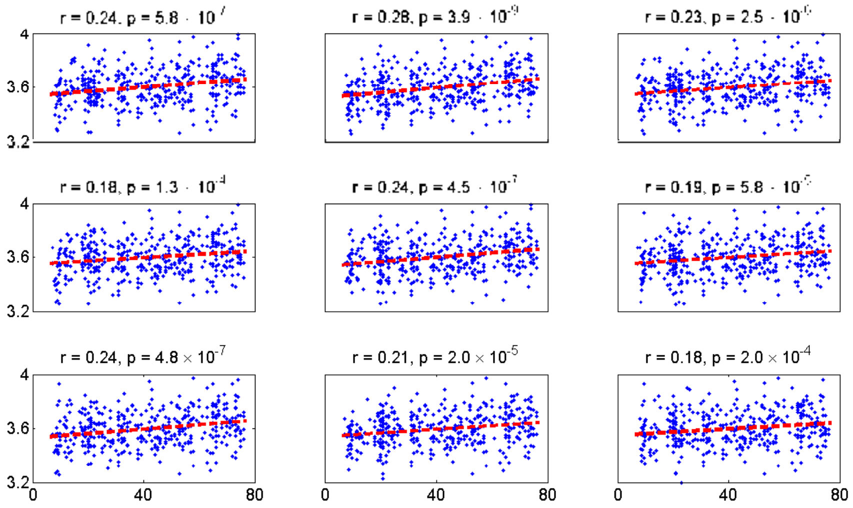

Figure S21

With different randomly selected subjects with flatting age distribution, the significant results were still present, functional entropy increased with age.

16 Understanding the functional entropy: a mathematical description

In this subsection, we present a mathematical description of the functional entropy defined in the main text. Let $x(t)$ be a resting fMRI time course of the brain. This is defined on a function space (with respect to time), Θ, which is a subspace of the Lebesgue function space $L^2(T, R)$, where T can be either the continuous time set $[0, S]$ or the discrete time set $\{1,2, \dots, S\}$. We assume that a probability measure, P, is defined on Θ with its σ-algebra $\mathcal{F}$ induced by the norm of $L^2(T, R)$. In the following, we need not know the explicit forms of the probability distributions of the random functions, to define our entropy. One particular fMRI time course from one of the regions of interest, which were considered, is regarded as a state point in the probability space $\{\Theta, \mathcal{F}, P\}$. Let $x(\cdot)$ and $y(\cdot)$ be two independent random time courses following the same distribution. Their correlation coefficient can be regarded as a functional with respect to $\Theta \times \Theta$:

$$\rho(x, y) = \frac{\frac{1}{S}\int_0^S (x(t)-\bar{x})(y(t)-\bar{y})dt}{std(x)std(y)} \quad (16)$$

with $\bar{x} = \frac{1}{S}\int_0^S x(t)dt$, $std(x) = \sqrt{\frac{1}{S}\int_0^S [x(t) - \bar{x}]^2 dt}$. The functional entropy we defined is actually that of the random (scale) variable $\rho(x, y)$ that is induced by the two independent random functions $x(\cdot)$ and $y(\cdot)$. Thus, we write the entropy as $H(\rho(x, y))$. The entropy of a region can be regarded as the specific conditional entropy of $\rho(x, y)$ when y is fixed, namely $H(\rho(x, y)|y = y(\cdot))$. The mean of a region's entropy can be regarded as the conditional entropy of $\rho(x, y)$ with respect to the random function $y(t)$, and we write this entropy as $H(\rho(x, y)|y)$.

To specify the meaning of the definition of functional entropy given, we restrict the function space Θ to all periodic functions with the same constant frequency, ω. Then $x(t)$ can be written as $x(t) = a \times \cos(\omega t + \varphi)$ and $y(t)$ can be written as $y(t) = b \times \cos(\omega t + \psi)$. For sufficiently large S, the mean of both x and y are approximately zero. Their correlation coefficient, as $S \to \infty$, becomes

$$\rho(x, y) = \frac{\frac{1}{2S}\int_0^S ab\times[\cos(2\omega t+\varphi+\psi)+\cos(\varphi-\psi)]dt}{ab\sqrt{\frac{1}{S^2}\int_0^S [\cos(\omega t+\varphi)]^2 dt \int_0^S [\cos(\omega t+\psi)]^2 dt}} \to \cos(\varphi - \psi). \quad (17)$$

Additionally, $\rho(x, y) = \cos(\varphi - \psi)$ also holds if S is an integer multiple of the period. Thus, the correlation coefficient between two periodic oscillations with the same period is the cosine of the phase difference. In this scenario, the function space can be embedded in a phase space (a one dimensional torus)

$\mathcal{S}^1$ (namely,$[0,2\pi]$). Let $\{\mathcal{S}^1, \mathrm{F}, \mathrm{P}\}$ be the probability space induced by $\{\Theta, \mathcal{F}, \mathrm{P}\}$, let φ and ψ be two independent random variables in it, and let $\mathrm{z} = \cos(\varphi - \psi)$. Then, the entropy of z is $\mathrm{H(z)}$, and the region's entropy is the specific conditional entropy, $\mathrm{H}(\mathrm{z}|\varphi = \varphi_0)$, and the mean regional entropy is the conditional entropy $\mathrm{H}(\mathrm{z}|\varphi)$. Note that in the definition of the entropy, the Lebesgue measure of $\mathrm{z} = \cos(\varphi - \psi)$ is the trivial one defined in [-1, 1], denoted by $\mathrm{m}_{cos}(dz)$. The difference between the entropy of z and its conditional entropy is clearly the mutual information between φ and z:

$\mathrm{I}(\mathrm{z}, \varphi) = \mathrm{H}(\mathrm{z}) - \mathrm{H}(\mathrm{z}|\varphi)$. (18)

Let $\theta = |\varphi - \psi|$, then, z is equivalent to θ, considering the torus $\mathcal{S}^1$ with $\theta \equiv 2\pi + \theta$. With a properly-defined Lebesgue measure of θ, i.e., the measure induced by that of z, namely $\mathrm{m}_{cos}(\cdot)$, the entropy and conditional entropy of θ then equal those of z. Let us pick the measures of these variables, in order to define the (relative) entropy. That is: First, pick a joint measure of (φ, ψ) (denoted by $\mathrm{m}_1(d\varphi, d\psi) = m(d\varphi)m(d\psi)$, a joint measure of two independent and identical measures, where $m(\cdot)$ is determined below), and a joint measure of $(\varphi, \varphi - \psi)$, denoted by $\mathrm{m}_2(d\varphi, d(\varphi - \psi))$, such that they are preserved through the transformation between them, (i.e., letting T be the transform from (φ, ψ) to $(\varphi, \varphi - \psi)$, then for any measurable set A in the space of $(\varphi, \varphi - \psi)$), $\mathrm{m}_2(A) = m_1(T^{-1}(A))$ holds); Second, pick the measure $(\varphi, \theta = |\varphi - \psi|)$ induced by $\mathrm{m}_1(d\varphi, d\psi)$ and denoted by $\mathrm{m}_3(d\varphi, d\theta)$; Finally, $m(\cdot)$ is chosen to guarantee that the embedded measure of θ equals to $\mathrm{m}_{cos}(dz)$. Thus, we have

$$2\mathrm{D}\big(\varphi \parallel \mathrm{m}(\cdot)\big) = \mathrm{D}\big(\varphi, \psi \parallel \mathrm{m}_1(\cdot,\cdot)\big) = \mathrm{D}\big(\varphi, \varphi - \psi \parallel \mathrm{m}_2(\cdot,\cdot)\big) \geq \mathrm{D}\big(\varphi, \theta \parallel \mathrm{m}_3(\cdot,\cdot)\big)$$
$$= \mathrm{D}(\theta|\varphi \parallel \mathrm{m}_3(\cdot,\cdot)) + \mathrm{D}(\varphi \parallel \mathrm{m}(\cdot)). \quad (19)$$

We conclude that $\mathrm{D}(\theta|\varphi \parallel \mathrm{m}_3(\cdot,\cdot)) \leq \mathrm{D}(\varphi \parallel \mathrm{m}(\cdot))$. This implies that the mean regional entropy is a lower-bound of the entropy of the phase random variable under the measure preserving transformation. In addition, if different measures were picked, there would be constant differences in the above inequality induced by the expectation of the derivative of different Lebesgue measures (see the relative entropy part above in 3.1).

In particular, if we pick the trivial measure on the torus, $\mathcal{S}^1$, and let $\mathrm{f}(\varphi)$ be the probability density function (pdf) ofφ, then after simple algebra, we have the conditional pdf of θ with respect to φ as

$\mathrm{p}(\theta|\varphi) = \mathrm{f}(\varphi - \theta) + \mathrm{f}(\varphi + \theta) \quad \pi \geq \theta \geq 0$. (20)

We then have

$\mathrm{H}(\theta|\varphi) = \mathrm{E}\{\log\{[\mathrm{f}(\varphi - \theta) + \mathrm{f}(\varphi + \theta)]\}$. (21)

17 References

1. Cover, T.M. & Thomas, J.A. *Elements of information theory* (Wiley-interscience, 2006).
2. Shannon, C.E. Prediction and entropy of printed English. *Bell System Technical Journal* **30**, 50-64 (1951).
3. Wehrl, A. General properties of entropy. *Reviews of Modern Physics* **50**, 221 (1978).
4. Barnes, C.A., Suster, M.S., Shen, J. & McNaughton, B.L. Multistability of cognitive maps in the hippocampus of old rats. *Nature* **388**, 272-275 (1997).
5. McIntosh, A.R., Kovacevic, N. & Itier, R.J. Increased brain signal variability accompanies lower behavioral variability in development. *PLoS computational biology* **4**, e1000106 (2008).
6. Vaseghi, S.V. *Advanced digital signal processing and noise reduction* (Wiley, 2008).
7. Vose, D. *Risk analysis: a quantitative guide* (Wiley, 2008).
8. Dobbs, D. Schizophrenia: The making of a troubled mind. *Nature* **468**, 154-156 (2010).
9. Deco, G. & Jirsa, V.K. Ongoing cortical activity at rest: criticality, multistability, and ghost attractors. *The Journal of Neuroscience* **32**, 3366-3375 (2012).
10. Friston, K.J., Harrison, L. & Penny, W. Dynamic causal modelling. *Neuroimage* **19**, 1273-1302 (2003).
11. Rocca, W., *et al.* Increased risk of parkinsonism in women who underwent oophorectomy before menopause. *Neurology* **70**, 200-209 (2008).
12. Pike, C.J., Carroll, J.C., Rosario, E.R. & Barron, A.M. Protective actions of sex steroid hormones in Alzheimer's disease. *Frontiers in neuroendocrinology* **30**, 239-258 (2009).
13. Cooper, C., Kuh, D., Egger, P., Wadsworth, M. & Barker, D. Childhood growth and age at menarche. *BJOG: An International Journal of Obstetrics & Gynaecology* **103**, 814-817 (1996).
14. Frackowiak, R.S. *Human brain function* (Academic Press, 2004).

18 Appendix: Matlab code of entropy

Code of the entropy of the whole brain:

```
% Entropy of the whole brain
function [Entropy_value]=Brain_Entropy(CorrMat)
% Input is the correlation matrix of the resting state BOLD signal.
% Output is the entropy value.
IntervalNumber=20;% Interval number in [-1,1]

% Extract the upper triangular matrix of CorrMat
CorrMatSize=size(CorrMat);
CorrMatLength=CorrMatSize(1);
CorrList=zeros(1,CorrMatLength*(CorrMatLength-1)/2);% Correlation list
k=0;
i=1;
while i<=CorrMatLength
    j=i+1;
    while j<=CorrMatLength
        k=k+1;
        CorrList(1,k)=CorrMat(i,j);
        j=j+1;
    end
    i=i+1;
end

% Entropy calculation
EntropyList=zeros(IntervalNumber,1);
i=1;
while i<=IntervalNumber
    TempPosi1=find((CorrList>-1+2/IntervalNumber*(i-1))& (CorrList<=-1+2/IntervalNumber*i));
    EntropyList(i,1)=length(TempPosi1);
    i=i+1;
end
EntropyList=EntropyList./sum(EntropyList);
TempPosi2=find(EntropyList~=0);
Entropy_value=-sum(EntropyList(TempPosi2).*log(EntropyList(TempPosi2)))/log(2);
```

Code of the entropy of the inter hemisphere:

```
% Entropy of the inter hemisphere (The odd rows of the correlation matrix is from the left hemisphere.)
function [Entropy_value]=Brain_Entropy_Inter(CorrMat)
% Input is the correlation matrix of the resting state BOLD signal.
% Output is the entropy value.
IntervalNumber=20;% Interval number in [-1,1]
```

```
% Extract the inter part of CorrMat
  CorrMatSize=size(CorrMat);
CorrMatLength=CorrMatSize(1);
CorrList=zeros(1,CorrMatLength*CorrMatLength/4);% Correlation list
k=0;
i=1;
while i<=CorrMatLength/2
    j=1;
    while j<=CorrMatLength/2
        k=k+1;
        CorrList(1,k)=CorrMat(2*i-1,2*j);
        j=j+1;
    end
    i=i+1;
end

% Entropy calculation
EntropyList=zeros(IntervalNumber,1);
i=1;
while i<=IntervalNumber
    TempPosi1=find((CorrList>-1+2/IntervalNumber*(i-1))& (CorrList<=-1+2/IntervalNumber*i));
    EntropyList(i,1)=length(TempPosi1);
    i=i+1;
end
EntropyList=EntropyList./sum(EntropyList);
TempPosi2=find(EntropyList~=0);
Entropy_value=-sum(EntropyList(TempPosi2).*log(EntropyList(TempPosi2)))/log(2);
```

Code of the entropy of the left hemisphere:

```
% Entropy of the Left hemisphere (The odd rows of the correlation matrix is from the left hemisphere.)
function [Entropy_value]=Brain_Entropy_Left(CorrMat)
% Input is the correlation matrix of the resting state BOLD signal.
% Output is the entropy value.
IntervalNumber=20;% Interval number in [-1,1]

% Extract the left part of CorrMat
CorrMatSize=size(CorrMat);
CorrMatLength=CorrMatSize(1);
CorrList=zeros(1,CorrMatLength*(CorrMatLength/2-1)/4);% Correlation list
k=0;
i=1;
while i<=CorrMatLength/2
    j=i+1;
```

```
    while j<=CorrMatLength/2
        k=k+1;
        CorrList(1,k)=CorrMat(2*i-1,2*j-1);
        j=j+1;
    end
    i=i+1;
end

% Entropy calculation
EntropyList=zeros(IntervalNumber,1);
i=1;
while i<=IntervalNumber
    TempPosi1=find((CorrList>-1+2/IntervalNumber*(i-1))& (CorrList<=-1+2/IntervalNumber*i));
    EntropyList(i,1)=length(TempPosi1);
    i=i+1;
end
EntropyList=EntropyList./sum(EntropyList);
TempPosi2=find(EntropyList~=0);
Entropy_value=-sum(EntropyList(TempPosi2).*log(EntropyList(TempPosi2)))/log(2);
```

Code of the entropy of the right hemisphere:

```
% Entropy of the Right hemisphere (The odd rows of the correlation matrix is from the left hemisphere.)
function [Entropy_value]=Brain_Entropy_Right(CorrMat)
% Input is the correlation matrix of the resting state BOLD signal.
% Output is the entropy value.
IntervalNumber=20;% Interval number in [-1,1]

% Extract the right part of CorrMat
CorrMatSize=size(CorrMat);
CorrMatLength=CorrMatSize(1);
CorrList=zeros(1,CorrMatLength*(CorrMatLength/2-1)/4);% Correlation list
k=0;
i=1;
while i<=CorrMatLength/2
    j=i+1;
    while j<=CorrMatLength/2
        k=k+1;
        CorrList(1,k)=CorrMat(2*i,2*j);
        j=j+1;
    end
    i=i+1;
end

% Entropy calculation
```

```
EntropyList=zeros(IntervalNumber,1);
i=1;
while i<=IntervalNumber
    TempPosi1=find((CorrList>-1+2/IntervalNumber*(i-1))& (CorrList<=-1+2/IntervalNumber*i));
    EntropyList(i,1)=length(TempPosi1);
    i=i+1;
end
EntropyList=EntropyList./sum(EntropyList);
TempPosi2=find(EntropyList~=0);
Entropy_value=-sum(EntropyList(TempPosi2).*log(EntropyList(TempPosi2)))/log(2);
```

Code of the entropy of all the brain regions:

```
% Entropy of all the brain regions
function [Entropy_Region_value]=Brain_Region_Entropy(CorrMat)
% Input is the correlation matrix of the resting state BOLD signal.
% Output is the entropy value of different regions.
IntervalNumber=20;% Interval number in [-1,1]

CorrMatSize=size(CorrMat);
CorrMatLength=CorrMatSize(1);% Region Number
Entropy_Region_value=zeros(CorrMatLength,1);
iRegionNumber=1;
while iRegionNumber<=CorrMatLength
% Extract the CorrList from CorrMat
CorrList=zeros(1,CorrMatLength-1);% Correlation list
k=0;
i=1;
while i<=CorrMatLength
    if i~=iRegionNumber
        k=k+1;
        CorrList(1,k)=CorrMat(i,iRegionNumber);
    end
    i=i+1;
end

% Entropy calculation
EntropyList=zeros(IntervalNumber,1);
i=1;
while i<=IntervalNumber
    TempPosi1=find((CorrList>-1+2/IntervalNumber*(i-1))& (CorrList<=-1+2/IntervalNumber*i));
    EntropyList(i,1)=length(TempPosi1);
    i=i+1;
end
EntropyList=EntropyList./sum(EntropyList);
```

```
TempPosi2=find(EntropyList~=0);
Entropy_Region_value(iRegionNumber,1)=-sum(EntropyList(TempPosi2).*log(EntropyList(TempPosi2))
)/log(2);
iRegionNumber=iRegionNumber+1;
end
```
